# Bricks and mortar of well-being: exploring the housing-health connection

**DOI:** 10.1186/s12889-023-16844-9

**Published:** 2023-10-03

**Authors:** Roshanak Mehdipanah, Lara C. Weinstein

**Affiliations:** 1 Department of Health Behavior & Health Education, University of Michigan School of Public Health, 1415 Washington Heights, SPH 1, Ann Arbor, MI 48109-2029 USA; 2 Department of Family and Community Medicine, Jefferson Medical College, Thomas Jefferson University, 1015 Walnut, Philadelphia, PA 19107 USA

## Abstract

Housing is a determinant of health. Increasing housing costs and worsening housing conditions have impacted populations worldwide. This Editorial aims to examine the dynamic connection between housing and health and the role of public health in addressing this relationship for a Collection launched at BMC Public Health on Housing as a determinant of health and well-being.

Housing is a determinant of health consisting of multiple dimensions including affordability and conditions or quality. Understanding the relationships between these dimensions and various health outcomes requires considerations of the various ecological levels including policy, community and individual levels that operate independently and jointly to influence population health. Furthermore, housing and health form a bidirectional relationship as health conditions can drive housing outcomes, highlighting the need to address housing and health inequities amongst diverse populations.

Much of the existing literature on housing and health has focused on conditions which refer to the overall state and quality of the house. This includes the structural integrity, maintenance, safety, sanitation and access to essential amenities like water, electricity and heating/cooling systems. These physical conditions have been linked to various physical and mental health outcomes [1]. For example, poor ventilation, mold and dampness have been associated with poor indoor air quality impacting respiratory conditions like asthma [2, 3]. Lack of proper heating or cooling can result in stress-related health issues, particularly among older populations [4]. Furthermore, houses without proper safety features like handrails and smoke detectors can increase the risk of injuries [5]. While housing repairs can address some of these issues, associated costs can be unaffordable resulting in greater insecurities.

Over the last two decades, more research has emerged examining the intersections of housing affordability and health, primarily driven by the economic recession of the late 2000s. Although the definition of housing affordability can vary by region and country, most definitions consist of the ability of a household to comfortably meet their housing costs without experiencing financial strain. For example, in the US, the Department of Housing and Urban Development defines housing as affordable if the household spends no more than 30% of their income on housing costs, including rent, mortgage, property taxes, utilities and other related costs [6]. Beyond the 30% threshold, housing is considered unaffordable, resulting in cost burden and placing the household at greater risk for housing instability. Unaffordable housing can impact access to food, transportation and medical care, all important factors for well-being. Correspondingly, medical debt, which is disproportionately experienced by marginalized populations, is widely recognized as a driver of housing insecurity [7]. Furthermore, the fear or experience of being evicted or foreclosed, along with frequent moving, doubling-up and homelessness, has been associated with negative health outcomes and interruptions to accessing care [8, 9].

Social conditions related to housing are a prominent determinant of health for populations experiencing medical or psychiatric related disability. Institutionalization and incarceration often replaces housing for people with serious mental illness and substance use disorders Permanent supportive housing (PSH), is defined by the US Department of Housing and Urban Development as a housing intervention which combines affordable housing assistance and supportive social services to help people with disabilities achieving housing stability [10]. These social supports can make the difference between a healthy and meaningful life in the community versus isolation in institutions or homelessness.

To address some of these housing issues, a multi-sectoral approach is needed. By involving multiple sectors, comprehensive and effectives solutions that address the root causes and underlying factors contributing to housing problems. Different sectors bring unique perspectives and expertise, and housing issues are rarely isolated and require combining expertise from various fields including urban planning, public health, economics and social work. An integrated approach can help address the diverse needs of communities, allowing for a focus on equity and social justice, ensuring the interventions consider specific needs and challenges faced by different groups. For example, people with complex medical, psychiatric, and substance use related co-morbidities are experiencing increasing rates of hospital admissions for substance use related medical conditions and are often unsheltered. Both healthcare systems and payers can develop, implement, and advocate for system level changes such as hospital support for temporary housing options.

As part of this collection on housing and health, we are seeking to present a diverse and informative range of articles that advance knowledge and foster innovative approaches to address and improve housing issues and promote better health outcomes worldwide. We are seeking articles that provide insight on emerging housing issues that intricately combine dimensions of affordability and living conditions, and their impact on health. We recognize that the location of the house also plays an important role on health. For example, housing within neighborhood with low pollution levels and minimal exposure to environmental hazards has been linked to reduced respiratory issues including asthma, while proximity to healthy food options and green spaces have been associated have been associated with reductions in chronic illness and stress-related disorders. However, for the purposes of this paper, we would like to focus on articles addressing the housing unit itself and not the surrounding areas, unless these factors are considered as moderators or mediators in the pathways to health.

We are also seeking articles that explore innovative housing solutions through a multisectoral approach, where collaboration among various fields, such as urban planning, public health, economics and social works, is employed to address housing issues comprehensively. Additionally, we welcome examples or case studies that highlight the involvement of the healthcare sector in funding or partnering for housing initiatives, showcasing successful models of collaboration. Furthermore, the edition aims to include global research that examines the relationship between housing and health, encompassing diverse geographical contexts and contributing to a broader understanding of the critical issue.

## Data Availability

Not applicable.
